# Effects of Samarium Doping on the Dielectric Properties of BaBi_2_Nb_2_O_9_ Aurivillius Ceramics

**DOI:** 10.3390/ma17204952

**Published:** 2024-10-10

**Authors:** Michał Rerak, Jolanta Makowska, Tomasz Goryczka, Beata Wodecka-Duś, Katarzyna Osińska, Grzegorz Tytko, Małgorzata Adamczyk-Habrajska

**Affiliations:** 1 Institute of Materials Engineering, Faculty of Science and Technology, University of Silesia, 75 Pułku Piechoty 1A, 41-500 Chorzow, Poland; michal.jan.rerak@gmail.com (M.R.); jolanta.makowska@us.edu.pl (J.M.); to-masz.goryczka@us.edu.pl (T.G.); beata.wodecka-dus@us.edu.pl (B.W.-D.); katarzyna.osinska@us.edu.pl (K.O.); 2 Faculty of Automatic Control, Electronics and Computer Science, Silesian University of Technology, 44-100 Gliwice, Poland; grzegorz.tytko@polsl.pl

**Keywords:** dielectric properties, BaBi_2_Nb_2_O_9_, samarium (Sm^3+^) doping

## Abstract

This study investigates the influence of samarium (Sm^3+^) doping on the structural, microstructural, mechanical, and dielectric properties of BaBi_2_Nb_2_O_9_ (BBN) ceramics. Using the solid-state reaction method, samples of BaBi_2-x_Sm_x_Nb_2_O_9_ with varying concentrations of Sm (*x* = 0.01; 0.02; 0.04; 0.06; 0.08; 0.1) were prepared. Thermal analysis, microstructure characterization via SEM and EDS, X-ray diffraction, mechanical testing, and dielectric measurements were conducted. The results revealed that increasing Sm^3+^ concentration led to the formation of single-phase materials with a tetragonal structure at room temperature. Mechanical properties, such as Young’s modulus and stiffness, improved with Sm doping, indicating stronger atomic bonding. Dielectric properties showed that low concentrations of Sm^3+^ slightly increased electrical permittivity, while higher concentrations reduced it. The presence of Sm^3^⁺ also affected the relaxor properties, evidenced by changes in the freezing temperature and activation energy. Overall, the study concludes that samarium doping enhances the structural and functional properties of BBN ceramics, making them promising candidates for high-temperature piezoelectric and dielectric applications. The findings provide valuable insights into tailoring ceramic materials for advanced technological applications.

## 1. Introduction

Although relaxor ferroelectrics have been studied extensively over the years, they remain a focus of ongoing research due to their significant technological potential and diverse applications. However, the microscopic mechanisms underlying their behavior are still not well understood. Relaxor ferroelectrics exhibit a diffuse phase transition, leading to a broadening of the maximum electric permittivity (ε_max_). Additionally, both this maximum and the temperature at which it occurs (T_m_) show frequency dispersion [1,2,3,4]. Most studies on relaxor ferroelectrics have focused on lead-based complex perovskites like PMN (lead magnesium niobate) and PLZT (lead lanthanum zirconate titanate), known for their high dielectric constants and significant electrostrictive effects [5,6,7,8,9,10]. However, concerns over lead’s toxicity have prompted the search for alternative materials [11,12]. While extensive research has been conducted on lead-based materials, there is less emphasis on relaxor ferroelectrics from other oxide families, particularly Bi-based layered perovskites. Bismuth-based layered perovskites (BLFs) are promising due to their excellent properties and potential for nonvolatile ferroelectric random-access memory (NVFRAM) and high-temperature applications [13,14,15]. These BLFs are of considerable interest for high-temperature piezoelectric devices owing to their high Curie temperature (T_C_), exceptional thermal stability, high resistivity, and outstanding fatigue resistance [16,17]. Furthermore, bismuth-layered Aurivillius oxides have been developed to rival PZT properties, enhancing the understanding and application of relaxor ferroelectrics. Thus, further studies on these alternative materials could greatly expand the potential uses of relaxor ferroelectrics.

The structure of bismuth-layered structure ferroelectric compounds can be described by the general formula ${\left(M_{2}O_{2}\right)}^{2+}{\left(A_{n-1}B_{n}O_{3m+1}\right)}^{2-}$. In this formula, A represents a 12-fold, coordinated cation such as Bi^3+^, Sr^2+^, Ba^2+^, and Ca^2+^ [18,19], while B is a 6-fold coordinated cation like Nb^5+^, Ta^5+^, W^6+^, and Mo^6+^ [19,20]. The place of the M cation in the oxygen layers is usually taken by bismuth ions [21]. The variable n is an integer or half-integer (ranging from 1 to 6) that indicates the number of (A_n−1_B_n_O_3n+1_)^2−^ octahedral layers situated between the (Bi_2_O_2_)^2+^ layers in the lattice structure of these compounds. For many years, it was believed that substituting the M ion in the (M_2_O_2_)^2+^ layers was impossible because these layers act as thermodynamic stabilizers of the structure [21]. However, recent studies by various research groups have demonstrated that it is at least partially possible to substitute ions of other elements, such as Pb^2+^, Sn^2+^, Sb^3+^, or Te^4+^, in the (M_2_O_2_)^2+^ layers of the Aurivillius structure [21,22,23,24,25,26].

BaBi_2_Nb_2_O_9_ (BBN) is a Bi-layered (n = 2) pseudo-perovskite Aurivillius ceramic. Extensive research has underscored their potential applications and advantages, positioning them as a significant focus in contemporary studies [27,28,29,30,31,32]. This compound crystallizes in either an orthorhombic or tetragonal structure and demonstrates a strong broadened ferroelectric–paraelectric transition between *T* = 100–150 °C, marked by relaxor behavior [21,33]. It is generally acknowledged that Bi_2_O_2_ layers play a crucial role in the electrical conductivity characteristics of bismuth-based layered structures [34]. Extensive research has been undertaken to improve their properties through the substitution of Bi^3+^ ions with alternative cations. Recently, there has been a notable surge in interest regarding the doping of trivalent rare earth ions in layered ferroelectric structures, primarily due to their significant effects on the physical properties [35].

The aim of the current study is to elucidate the impact of Sm^3^⁺ doping on the structural, thermal, mechanical, and electrical behaviors of BBN ceramics. We present the results of a comprehensive analysis, including microstructural characterization, differential thermal analysis (DTA), X-ray diffraction (XRD), mechanical property evaluation, and electrical permittivity measurements. The goal of these extensive analyses is to understand the role of bismuth layers in shaping the properties of perovskite-like compounds, which will undoubtedly contribute to optimized performance in specific applications of these materials in the electronics industry.

## 2. Materials and Methods

The research material was produced using conventional technology, i.e., solid-phase synthesis reaction and free sintering in an air atmosphere. The stage that started the ceramics production technology was weighing the substrates in stoichiometric quantities, i.e., barium carbonate (BaCO_3_—Sigma-Aldrich, St. Louis, MO, USA, 98.5%) bismuth (III) oxide (Bi_2_O_3_—Aldrich, St. Louis, MO, USA, 99.9%), niobium (V) oxide (Nb_2_O_5_—Aldrich, St. Louis, MO, USA, 99.9%), and samarium (III) oxide (Sm_2_O_3_—Aldrich, St. Louis, MO, USA, 99.9%). The synthesis reaction of the reference composition (BaBi_2_Nb_2_O_9_) and samarium doped with ions took place according to Equations (1) and (2):

(1) $${\mathrm{BaCO}}_{3}+{\mathrm{Bi}}_{2}O_{3}+{\mathrm{Nb}}_{2}O_{5}\to {\mathrm{BaBi}}_{2}{\mathrm{Nb}}_{2}O_{9}+{\mathrm{CO}}_{2}↑$$

(2) $$2{\mathrm{BaCO}}_{3}+x{\mathrm{Sm}}_{2}O_{3}+\left(2-x\right){\mathrm{Bi}}_{2}O_{3}+2{\mathrm{Nb}}_{2}O_{5}\to 2{\mathrm{BaBi}}_{2-x}{\mathrm{Sm}}_{x}{\mathrm{Nb}}_{2}O_{9}+2{\mathrm{CO}}_{2}↑$$

The stoichiometric mixture of oxides and carbonates was ground in a planetary ball mill for *t* = 24 h (with 97% ethyl alcohol, POCH CZDA, Gliwice, Poland). The homogenized substrate mixture was air-dried for *t* = 48 h. A small amount of powder was poured off for thermal analysis. The next stage of the technology was the formation of moldings in the shape of cylindrical disks with a diameter of d = 25 mm using a hydraulic press at a pressure of *p* = 300 MPa. The synthesis process took place at a temperature of *T* = 950 °C for *t* = 4 h using the solid phase synthesis reaction method. A small amount of powder was again prepared for thermal analysis after synthesis. After completing the synthesis process, the compacts were crushed in a mortar, then wet ground again for *t* = 24 h and air-dried for *t* = 48 h. The ceramic powder produced in this way was pressed under a pressure of *p* = 600 MPa, using a hydraulic press, into discs with a diameter of d = 10 mm. The material prepared in this way was subjected to final free sintering in an air atmosphere at a temperature of *T* = 1100 °C for *t* = 2 h.

Differential thermal analysis (DTA) and thermogravimetric analysis (TG/DTG) were used to determine the thermochemical properties of the obtained powder. Simultaneous measurements were executed by heating the dried powders in the air at *T* = 10 °C/min to *T* = 1050 °C. The MOM Q-1500D (system Paulik-Paulik-Erdey, Hungary, Budapest) thermal analyzer was used for the studies.

The microstructure and chemical composition of the ceramics were analyzed using a JSM-7100F scanning electron microscope (SEM) paired with a NORAN Vantage energy dispersive spectrometer (EDS) (Tokyo, Japan). The process involved randomly selecting multiple fields across the surface of the samples for image capture, with both qualitative and quantitative chemical analyses conducted using X-ray microanalysis. The grain size was analyzed based on microstructural SEM images using the linear intercept method with the image analysis software ImageJ (ImageJ 1.37v, LOCI, University of Wisconsin—Madison, WI, USA).

The phase analysis and structural characterization of the material were carried out using an X’Pert PRO X-ray diffractometer from PANalytical (Almelo, Netherlands). All diffractograms were measured in the angular range of 2θ from 5° to 140° in the step scan mode with a measuring 2θ step of 0.04° and time of 3 s. An X-ray tube with a copper anode was applied with K_α1_ and K_α2_ radiation and a wavelength of 1.540601 Å and 1.54443 Å, respectively. Phase identification was carried out based on. the ICDD-PDF4 international database.

Mechanical tests using ultrasound were carried out based on the UZP-1 apparatus (INCO-VERITAS, Warsaw, Poland) using ultrasonic heads with a frequency of *f* = 10 MHz to measure the speed of longitudinal waves and oil as a coupling medium. Transducers with a frequency of *f* = 2 MHz were used to measure transverse waves, and Canada balsam acted as the coupling medium. The tests were performed on disc-shaped samples with diameters of d = 8–9.4 mm and a thickness h = 2.5 mm. The measurement consisted of determining the propagation times of both transverse and longitudinal sound waves along the disc’s diameter and height. Each time, the first impulses were measured, and, when possible, the successive echoes. The velocities of the transverse and longitudinal waves were calculated as the ratio of the sample’s thickness to the corresponding transit time. The accuracy of the ultrasonic wave velocity measurement was ± 10 m/s [36,37].

A computerized automated system utilizing the precision LCR meter Agilent E4980A (Santa Clara, CA, USA). was employed to measure the temperature-dependent permittivity within the *f* = 0.1 kHz to *f* = 1 MHz frequency range. Samples used for dielectric measurements were disk-shaped, with a surface area of 1 cm^2^ and a thickness of 1 mm. Before actual measurements, the samples were subjected to stress relief annealing at a temperature of T = 400 °C for t = 0.5 h and were coated with silver electrodes using a silver paste (P-120, supplier: Polish State Mint, Warsaw, Poland), followed by reannealing at a temperature of T = 600 °C for t = 0.5 h.

## 3. Results and Discussion

### 3.1. Thermal Analysis

**Figure 1:**
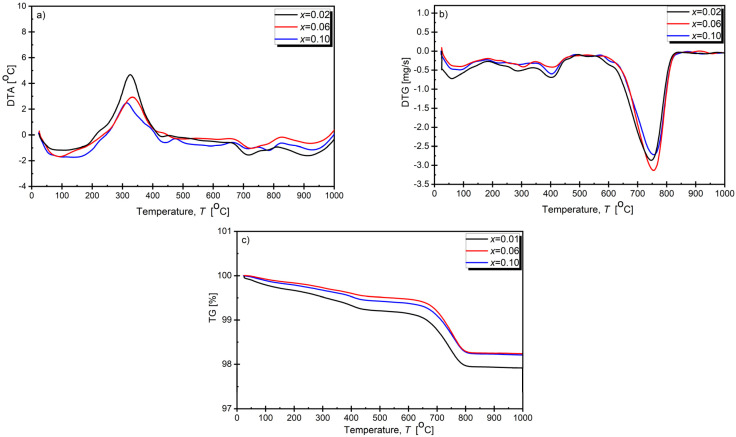
Results of thermal analysis of stoichiometric substrate mixture BaCO_3_+(1−x)Bi_2_O_3_+Nb_2_O_5_+*x*/2Sm_2_O_3_ of BaBi_2_Nb_2_O_9_ material doped with Sm^3+^ ions. (**a**) DTA—differential thermal analysis, (**b**) DTG—derivative thermogravimetry, (**c**) TG—thermogravimetric analysis.

Thermal analysis tests were performed before and after synthesis for all dopant concentrations. Figure 1 shows selected courses of differential thermal analysis (DTA), thermogravimetric analysis (TG), and differential thermogravimetry (DTG) for compositions with mole fractions of *x* = 0.02, *x* = 0.06, and *x* = 0.10 before synthesis.

Three temperature ranges were observed in which mass loss occurred. The first range included the temperature Δ*T*_1_ = (20–180) °C. The mass loss was at the level of ∆*m*_1_ = 0.3% and was related to the moisture evaporation from the samples. It corresponds to the maxima on the DTG curve. Moreover, endothermic maxima were observed on the DTA curve in this temperature range. The second mass loss (amounting to Δ*m*_2_ ≈ 0.5% in the temperature range Δ*T*_2_ = (180–460) °C) resulted from the transition of the α-Bi_2_O_3_ phase into the γ-Bi_2_O_3_ phase. It corresponds to the maximum on the DTG curve at around *T* = 380 °C and the exothermic maximum at the DTA curve at around *T* = 320 °C. The second mass loss discussed can be explained by the formation of oxygen vacancies, as postulated by the authors of [38] based on thermogravimetric analysis and observations using transmission electron microscopy. During heating, as the transition from the α phase to the γ phase occurred, the oxide composition changed from Bi_2_O_3_ to Bi_2_O_2.978_. The loss of oxygen is compatible with the minimum observed on the DTG curve. The third largest mass loss (Δmv ≈ 1%) was recorded for the temperature range of Δ*T*_3_ = (460–800) °C. It can be attributed to barium carbonate’s thermal decomposition, the release of carbon dioxide CO_2_, and a slight evaporation of bismuth(III) oxide [39,40,41,42,43]. Above the temperature *T* = 920 °C, no mass losses or corresponding energy effects were observed on the DTA curve, indicating that no chemical reactions occurred above this temperature. Based on the results of the thermal analysis of the initial BaBi₂Nb₂O₉ precursors, it was established that the minimum temperature for synthesizing a mixture of the initial components using this method should be at least 950 K, above which no anomalies on the DTA or TG curves are observed [44,45]. Moreover, this statement is consistent with the synthesis temperature values proposed by other authors [29,30,46].

### 3.2. X-ray Diffraction

**Figure 2:**
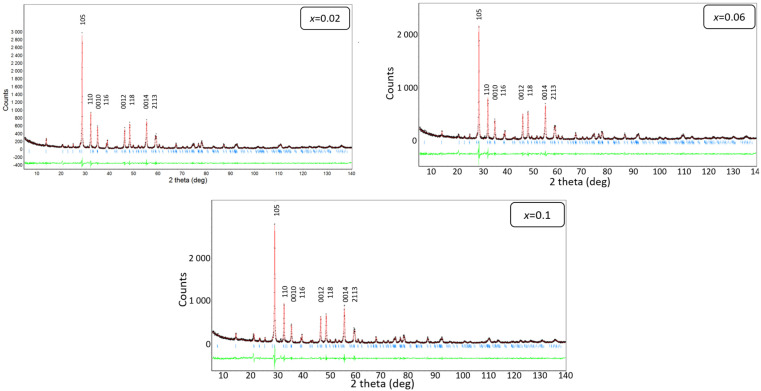
Graphical examples of the Rietveld refinement done for BaBi_2_Nb_2_O_9_ ceramics doped with Sm^3+^ ions.

The X-ray studies conducted were aimed at identifying the crystalline structure and phase composition of the ceramics. Detailed analysis of the results allowed for the determination of the influence of the doping element on the elementary cell parameters. First, the qualitative phase analysis was carried out. The intensity and position of the diffraction lines from the experimental diffractogram were compared to the standard from the international ICDD database. It was found that the BaBi_2_Nb_2_O_9_ phase was the only one present in the sample (the JCPDS card No. 00-12-0403). In the next step of analyzing the X-ray study results, the Rietveld method [47] was employed to determine the unit cell parameters (a, b, and c) and volume (V). The crystallographic data from the card 00-12-0403 were used for the built a starting model of the crystal structure. The graphical examples of the results are presented in Figure 2. The experimental diffraction pattern forms a black dot curve, whereas the calculated pattern is marked on the red line. The positions of the reflections are marked with blue bars, while the differential curve (green line) shows the differences between the experimental and calculated values—it represents the graphical quality of the refinement. It is worth mentioning that the reliability factors—used to assess the quality of fitting—revealed relatively low values. The R_p_ coefficient was not higher than 5.2, while the R_wp_ was 7.4 with 4.7 for R_exp_ [47].

The obtained ceramics BaBi_2_Nb_2_O_9_ clearly showed a typical crystal lattice characteristic for perovskites. Its symmetry can be described by the space group *I 4*/*mmm*. In order to compare the impact of samarium addition on the crystal structure, the parameters of the unit cell obtained from the calculations were compared with one obtained in our first work [48]—Table 1. As can be seen, the undoped crystal lattice revealed a higher value than the calculated ones. The data analysis in Table 1 indicates that the unit cell parameters of the doped ceramics showed a decreasing trend with increasing samarium concentration. The sample containing the maximum investigated concentration of samarium ions is an exception to this rule. The dominant decreasing tendency can be explained by the difference in the ionic radii of samarium and barium ($r_{{\mathrm{Sm}}^{3+}}=0.93\cdot 10^{-10}\;m,\;r_{{\mathrm{Ba}}^{2+}}$= 1.36·10^−10^ m and $r_{{\mathrm{Bi}}^{3+}}$ = 0.75·10^−10^ m [49]. Consequently, the volume of the unit cell also changed—its value, depending on the increase in Sm, decreased from 398 Å^3^ to 395 Å^3^. This supports the findings that the unique structure of layered perovskites, especially the presence of bismuth–oxygen layers, inhibits major alterations in the crystal lattice [50].

### 3.3. Analysis of Microstructure and Chemical Composition

**Figure 3:**
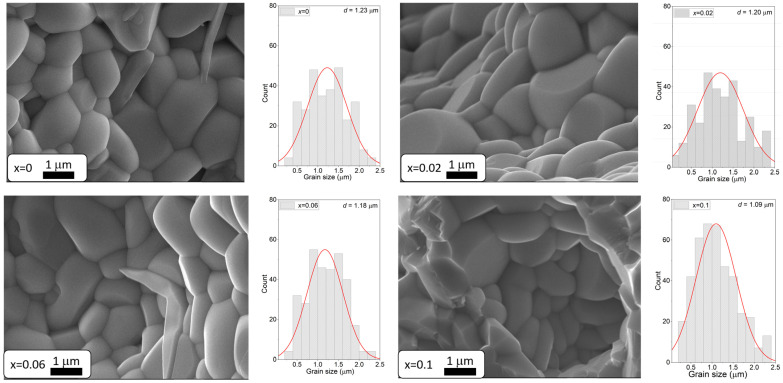
SEM image of BaBi_2_Nb_2_O_9_ ceramics doped with Sm^3+^ at concentrations of *x* = 0, *x* = 0.02, *x* = 0.06, and *x* = 0.1, magnified 15,000×. Next to it are grain size distribution diagrams.

Images of the microstructure of BaBi_2_Nb_2_O_9_ ceramics doped with Sm^3+^ for various dopant concentrations are shown in Figure 3.

The SEM image analysis of BaBi_2_Nb_2_O_9_ ceramics doped with samarium ions revealed that a higher dopant concentration (Sm^3+^) positively affected the material’s microstructure. The material exhibited a finer and more uniform grain structure, with an increase in well-developed grains. As the samarium content increased, the typically angular grain shapes became more rounded. Furthermore, the grain thickness was noticeably smaller compared to the other dimensions, reflecting the characteristic Aurivillius structure. This phenomenon is attributed to the weaker forces that promote the growth of crystallites along the c-axis, which runs perpendicular to the plate surface, as opposed to the stronger forces acting along the a and b axes [51]. The authors of the study [52] also reported comparable findings, noting that an increase in dopant levels influences the dimensions and morphology of grains. The modification of the base ceramic with samarium ions also contributed to a slight decrease in the average grain size, which changed from 1.23 for pure ceramics to 1.09 for ceramics doped with x = 0.1. The obtained results are consistent with the observations of the authors [31] and confirm the positive influence of samarium ions on the sinterability of bismuth-layered structure materials.

**Figure 4:**
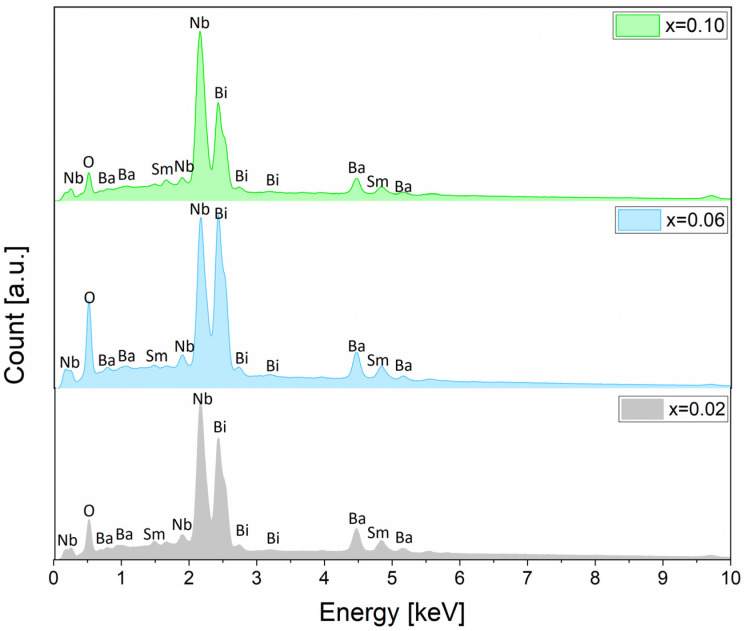
EDS spectrum of BaBi_2_Nb_2_O_9_ ceramics doped with Sm^3+^ ions.

The analysis of the chemical composition in the micro-areas of the tested samples was performed using an X-ray microanalyzer (EDS). The qualitative and quantitative composition of chemical elements constituting the tested material was determined. Examples of EDS spectra of BaBi_2_Nb_2_O_9_ ceramics doped with Sm^3+^ ions are shown in Figure 4.

The quantitative analysis of the chemical composition involved examining 50 randomly chosen micro-areas for each ceramic material and was aimed at determining the degree of deviation of the actual element content from the assumed theoretical stoichiometry. Average values were then calculated. The dispersion between the average percentage contents of individual components of the discussed compounds and the assumed theoretical stoichiometry was small and within the error limits of the method used. For example, the theoretical stoichiometric composition of the BBN ceramics doped with = 0.06 Sm^3+^ ions expressed as weight percentages relative to the oxides constituting it was BaO—21 wt%, Bi_2_O_3_—50.4 wt%, Nb_2_O_5_—27.8 wt%, and Sm_2_O_3_—0.8 wt%. The composition of the produced ceramics was BaO—22.4 wt%, Bi_2_O_3_—51.7 wt%, Nb_2_O_5_—30.1 wt%, and Sm_2_O_3_—0.8 wt%. It can be concluded that the manufactured ceramics maintained the assumed chemical composition. This proves that the technology used allowed for the production of BaBi_2−x_Sm_x_Nb_2_O_9_ materials, which are homogeneous in terms of chemical composition and are characterized by maintaining stoichiometry.

### 3.4. Mechanical Properties

Measurements of the propagation speed of transverse and longitudinal ultrasonic waves were carried out for all discussed compositions of the tested ceramics.

Measurements of longitudinal and transverse velocities were carried out in two directions: parallel and perpendicular to the direction of the pressing of the sample during the technological process. The obtained results differ slightly. Formula (3) was used to calculate the anisotropy A [53].

(3) $$A=\frac{100\%\cdot \left(V_{L1}-V_{L2}\right)}{V_{L1}}$$

where:

*V_L_*_1_—the average velocity of the longitudinal ultrasonic wave measured perpendicular to the sample’s pressing direction;
*V_L_*_2_—the average velocity of the longitudinal ultrasonic wave measured parallel to the sample’s pressing direction.

A similar methodology was employed for transverse waves propagating through the material under investigation. The determined anisotropy was of the order of 3%. In accordance with the methodology proposed by the authors of [53], the averaged velocities of both transverse and longitudinal ultrasonic waves were utilized for further calculations (Table 2).

The data presented in the table indicate a significant increase in the values of both longitudinal and transverse wave propagation speeds, which suggests a weakening of disturbances in the propagation of sound waves within the material. This fact can be linked to a reduction in defects caused by the modification of the base material with samarium ions [55,56]. The determined values of longitudinal and transverse ultrasonic wave propagation speeds in the tested ceramic materials allowed for the calculation of the Young’s modulus (*E*), stiffness modulus (*G*), and Poisson’s ratio (*μ*) (Table 3).

The recorded outstanding increase in mechanical properties is characteristic of the substitution of samarium ions in the (Bi_2_O_2_)^2+^ sublattice. A similar effect was noted by the authors of works [58,59]. Specifically, by modifying SrBi_4_Ti_4_O_15_ ceramics with Sm^3+^ ions in amounts of x = (0.0–0.75), they achieved an increase in the Young’s modulus from approximately 130 GPa to 155 GPa. More interestingly, a further increase in the concentration of samarium ions resulted in a decrease in the value of the Young’s modulus, which is consistent with the results presented in this paper. Such a significant improvement in the material’s mechanical properties can be explained by the crucial role of samarium ions in controlling bismuth and oxygen losses, which in turn leads to the previously mentioned reduction in defect concentration and, consequently, an enhancement in its mechanical properties.

### 3.5. Dielectric Properties

**Figure 5:**
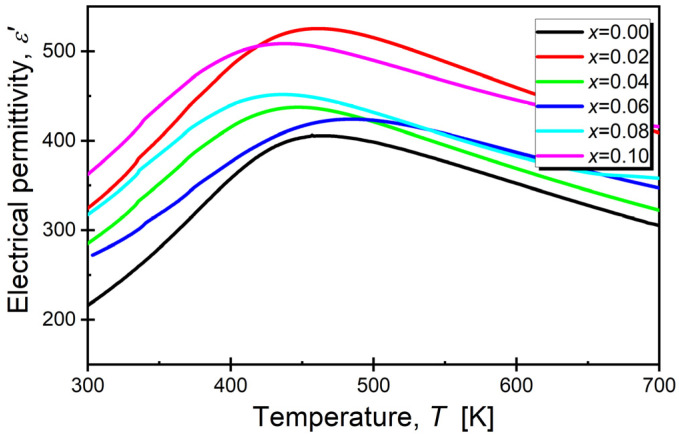
Temperature dependence of the real part of electric permittivity, with BaBi_2_Nb_2_O_9_ ceramics modified with Sm^3+^ ions obtained for a measuring field frequency equal to 100 kHz.

The temperature variations in dielectric permittivity obtained for a measuring field frequency equal to *f* = 100 kHz are shown in Figure 5.

Modification with samarium ions induced significant changes in the shape of the presented curves, primarily caused by alterations in the dielectric permittivity values compared to the reference sample. The maximum value of dielectric permittivity (ε_max_) initially increased for the two lowest concentrations, then decreased. However, it remained higher than the value observed in the reference sample (Table 4). The changes in dielectric permittivity can be attributed to the observed changes in unit cell volume. Specifically, an increase in lattice parameters led to greater polarizability, resulting in a higher dielectric constant. Similar behavior was reported by the authors of the following papers: [60,61]. For dopant concentrations greater than 0.02, a gradual decrease in the T_m_ temperature was observed. This can be explained as follows: The introduction of dopant ions increased disorder and generated additional local disturbances in the crystalline structure of the material, which in turn led to a reduction in the ordering of the crystal lattice, weakening long-range ferroelectric interactions and facilitating the transition to the paraelectric state, consequently resulting in the lowering of the T_m_ temperature.

**Figure 6:**
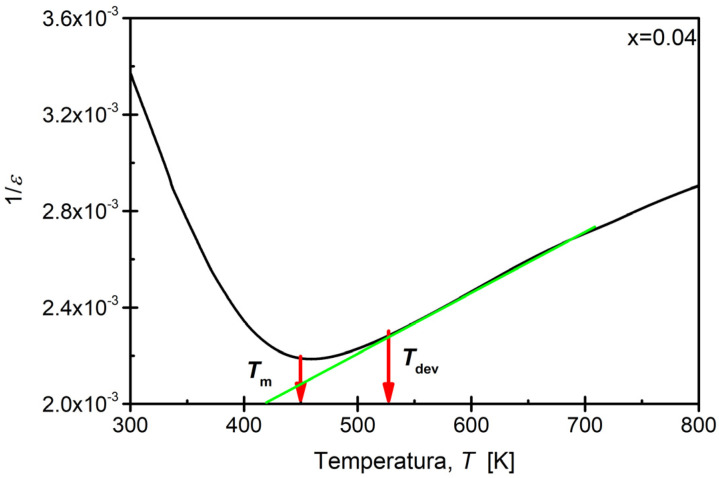
The temperature dependence of the inverse of the real part of the electrical permittivity determined for the cooling process in a measurement field with a frequency of f = 100 kHz for the BaBi_2_Nb_2_O_9_ ceramic modified with samarium ions for concentrations of x = 0.04.

Analyzing the shape of the maxima in the *ε*(T) dependencies revealed a substantial widening, which is a characteristic feature of BaBi_2_Nb_2_O_9_ ceramics, indicating the broadening of the phase transition. Such an effect is present in materials with a highly defective crystal lattice, where local charges exist, promoting the formation of randomly distributed electrostatic fields [21,62,63]. The strong broadening of the phase transition precludes the application of the classical Curie–Weiss law over a wide temperature range above the transition temperature *T*_m_, up to the deviation temperature *T*_dev_ [64]. Approximate values of the temperature *T*_dev_ were determined based on the dependence of 1/ε(T). The example of such dependence for ceramic modified with samarium ions for concentrations of *x* = 0.04 is presented in Figure 6.

**Figure 7:**
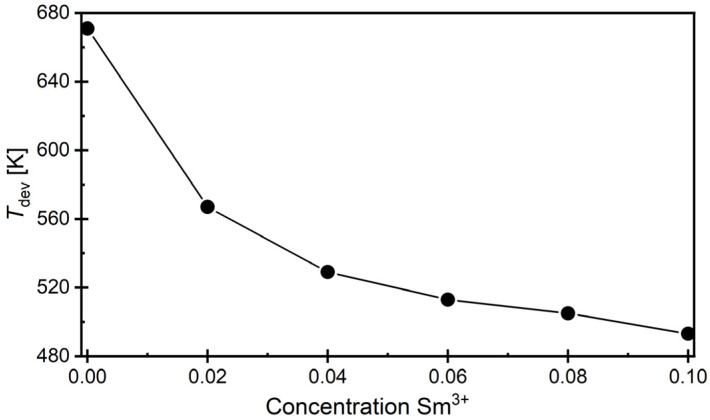
Dependence of the temperature *T*_dev_ (starting from which the classical Curie–Weiss law is applied) on the concentration of samarium admixture.

It was observed that an increase in the modifier concentration resulted in a shift in the discussed temperature towards lower values (Figure 7), leading to a significant narrowing of the temperature range *T*_m_–*T*_dev_, where the applicability of the modified Curie–Weiss law is justified (from *T* = 215 K for the reference ceramic to *T* = 58 K for ceramics containing *x* = 0.10 of Sm^3+^ substituted in bismuth-oxide layers, respectively).

In the temperature range of *T*_m_–*T*_dev_, the dependence of 1/*ε*(*T*) was described by the following modified Curie–Weiss law (4) [65,66,67]:

(4) $$\frac{1}{\varepsilon^{\prime }}-\frac{1}{\varepsilon_{max}^{\prime }}=\frac{{\left(T-T_{m}\right)}^{\gamma }}{C}$$

where:

*ε_max_*—maximum value of electric permittivity;
*C*—the Curie-like constant;
*γ*—the diffuseness parameter.

**Figure 8:**
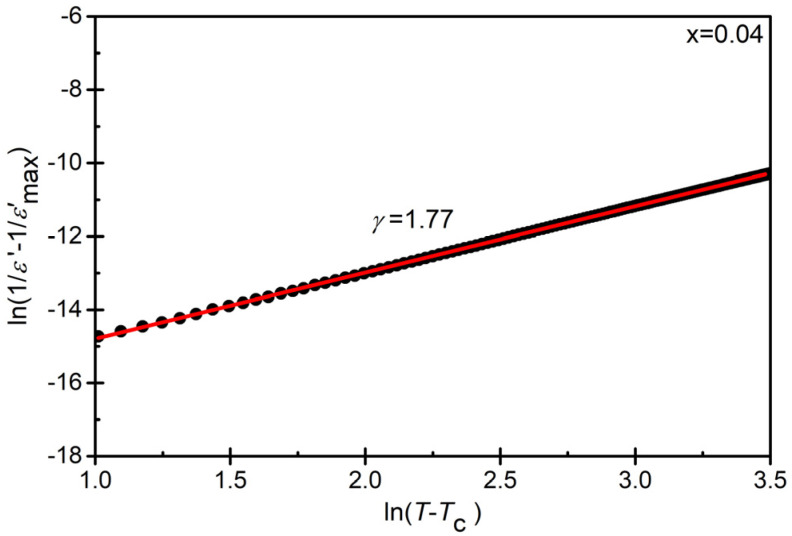
Graph of the dependence of ln (1/*ε* − 1/*ε_max_*) as a function of ln (*T* − *T*_m_)) for BBN ceramics doped with samarium in the amount of *x* = 0.04.

The fitting of experimental data to Equation (1) allowed for determining the diffuseness parameter γ (Table 5). An example of such fitting is presented in Figure 8 for concentrations of *x* = 0.04.

The data presented in Table 6 indicate an increase in the value of the parameter γ. This fact can be explained in the following way: Although no macroscopic phase segregation existed in the studied ceramic materials, chemical heterogeneity on a nanoscale cannot be excluded. Naturally, regions with different concentrations of the individual ions forming the compound will exhibit different nucleation temperatures for the polar phase. Locally, a phase transition will occur from a phase of higher symmetry to a phase of lower symmetry (hence, macroscopically, the symmetry will be broken). So-called polar nanoregions (PNRs) will emerge. The consequence of this state of affairs is a broadening of the temperature dependence peak of dielectric permittivity. In other areas, polar correlations are strongly diminished, and polar regions are less likely to nucleate. This leads to a redistribution of the charges and the local formation of charge centers. These are the source of local random fields, whose quenched spatial fluctuations act as pinning centers for the thermally fluctuating polarization [68]. The results presented in this publication indicate that the described scenario occurs to a moderate extent in pure BaBi₂Nb₂O₉ ceramics. The value of the γ parameter is at the level of 1.45. The introduction of another type of ion—the samarium ion—into the crystal structure resulted in an increase in disorder and the generation of additional mechanical stresses, ultimately leading to an increase in the diffuseness parameter γ. Such behavior can be attributed to the weakening of long-range ferroelectric interactions and high internal stress [67,69,70,71].

**Figure 9:**
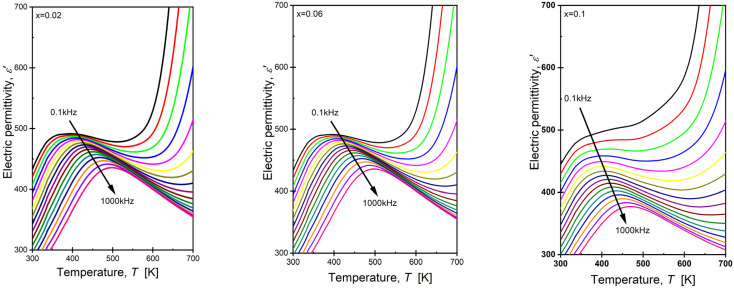
Temperature dependence. The real part of the electric permittivity component of BaBi_2_Nb_2_O_9_ ceramics doped with Sr^3+^ ions for the measurement field in the range f = 0.1 kHz–1 MHz (The arrow in the figures shows the direction of increasing frequency of the measurement field).

The undoped ceramic BaBi_2_Nb_2_O_9_ was characterized by strongly developed relaxor properties, manifesting, among other things, as a decrease in the maximum value of the real part of the electrical permittivity with an increase in the frequency of the measuring field [46,49,72]. The increase in this frequency simultaneously resulted in the shifting of the temperature *T*_m_ towards higher values. In the case of ceramic materials doped with samarium ions, the increase in the diffuseness parameter indicated an increase in the disorder of the crystal lattice. This phenomenon is typically associated with intensified properties typical for ferroelectric relaxors. To confirm these assumptions, the next step in the research was to determine the temperature-dependent characteristics of the real part of the electrical permeability as a function of the frequency of the measuring field. Sample dependence for the ceramic material BBN with samarium ions is presented in Figure 9.

The presented curves exhibited strong frequency dispersion characteristics of ferroelectric relaxors. The observed broad maximum decreased with the increase in the frequency of the measuring field, and the temperature of its occurrence shifted towards higher values. Widely recognized measures of the frequency dispersion of electrical permittivity and the temperature *T_m_* are Δ*ε_max_* and Δ*T_m_*, defined as follows in Equations (5) and (6) [73]:

(5) $$\Delta \varepsilon_{max}=\varepsilon_{max1kHz}-\varepsilon_{max1MHz}$$

(6) $$\Delta T_{m}=T_{m1MHz}-T_{m1kHz}$$

In the reference material, these parameters took on values of Δ*ε_max_* = 68.0 and Δ*T_m_* = 92.59 K [48]. The Δ*T_m_* value is especially noteworthy, as it exhibited significantly higher values compared to classical relaxors such as PMN or PLZT 8/65/35, where Δ*T_m_* is equal to 20 K [74] and 25 K [70], respectively. Table 6 presents these parameters’ values for BaBi_2_Nb_2_O_9_ ceramic modified with samarium ions.

The degree of temperature dispersion changes Δ*T*_m_ was abrupt and unordered, without a noticeable upward or downward trend. A more organized decreasing tendency was observed in the case of changes in the degree of dispersion of the maximum value of electrical permittivity.

The shape of the dependencies of *f*(*T_m_*) for all discussed ceramic points indicates that they can be well fitted with the Vogel–Fulcher law, originally derived for the spin-glass systems [75] (7):

(7) $$f=f_{o}exp\left[-\frac{E_{a}}{k_{B}\left(T_{m}-T_{f}\right)}\right]$$

where $f_{o}$ is the pre-exponential factor, *E_a_* is the activation energy related to the mechanism of dipole moment reorientation, *k_B_* is the Boltzmann constant, and *T_f_* is the characteristic of the freezing temperature.

**Figure 10:**
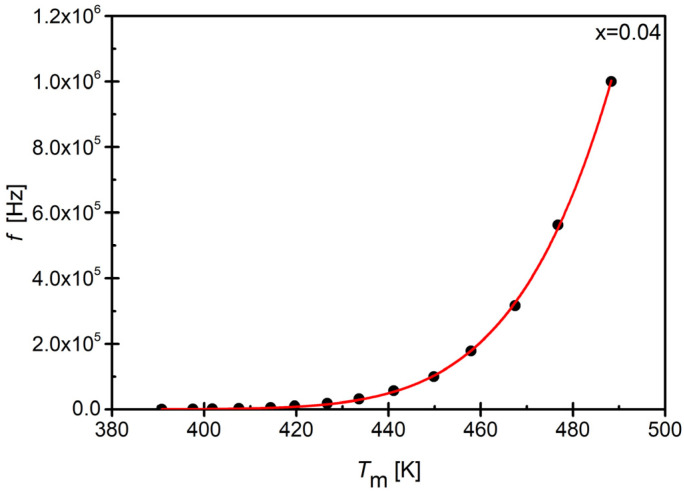
An exemplary dependence of *f*(*T*_m_) obtained for BBN ceramic doped with samarium at a concentration of x = 0.04. The red line indicates the fitting to the Vogel–Fulcher equation.

The example of the discussed function is presented in Figure 10 as an example.

**Figure 11:**
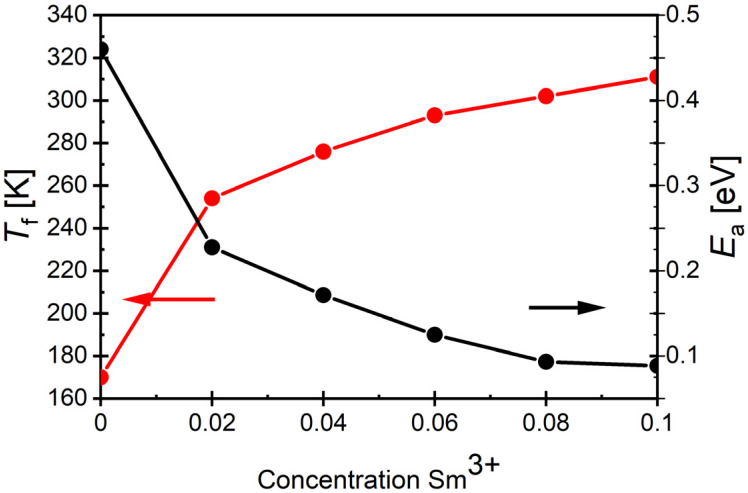
Dependence of the freezing temperature *T*_f_ and the activation energy *E*_a_ on the concentration of samarium admixture.

The conducted fitting experiments showed that the mentioned law described the experimental data very well. This fact allowed for the determination of parameters related to the behavior typical of ferroelectric relaxors, namely, the freezing temperature *T*_f_, where fluctuations in growing polar regions vanish and the system enters to the so-called polar glass state, as well as the activation energy E_a_, which is associated with the reorientation mechanism of dipole moments [76,77]. The determined values, obtained for the discussed ceramic materials as a function of the concentration of samarium ions, are presented in Figure 11.

The value of the activation energy *E*_a_ drastically decreased, and the freezing temperature *T*_f_ shifted towards higher values.

## 4. Conclusions

The main goal of this work was to present the impact of samarium doping introduced to the bismuth-oxide layers (Bi_2_O_2_)^2+^ on the microstructure, crystal structure, mechanical, and dielectric properties of BaBi_2_Nb_2_O_9_ ceramics. The obtained ceramic materials are characterized by a tetragonal structure at room temperature, with the I4/mmm space group, confirmed by X-ray analysis. Examination of the results revealed that adding a minor quantity of samarium did not alter the compound’s crystal structure. X-ray phase analysis showed that the materials produced are characterized by single-phase grains of the crystalline phase at room temperature. Microstructure analysis, performed using a scanning electron microscope, showed that an increase in the concentration of Sm^3+^ ions favors the formation of a fine-grained, homogeneous structure. The resulting grains are better developed and take on rounded shapes. The size of the grains decreases with an increase in samarium admixture. Moreover, the mechanical quality of the doped materials improves, as indicated by the results of mechanical tests. Specifically, under the influence of the admixture, the values of the Young’s modulus (E) and the stiffness modulus (G) increase. A slight addition of samarium ions (up to x = 0.02) also results in a slight increase in electrical permittivity, correlated with improved dielectric properties and a simultaneous increase in the blurring parameter. The presence of samarium ions also causes changes in quantities describing the relaxor properties of ceramics, such as freezing temperature and activation energy. The increase in T_f_, coupled with a simultaneous decrease in the temperature T_dev_, leads to a significant narrowing of the region where properties typical of ferroelectric relaxors occur, with an increase in the concentration of samarium ions. In summary, it can be concluded that the increasing concentration of samarium ions gradually leads to the disappearance of features typical of ferroelectric relaxors, while simultaneously narrowing the temperature range of their occurrence. The presented studies demonstrated the crucial role of (Bi_2_O_2_)^2+^ layers in shaping the properties of BaBi_2_Nb_2_O_9_ ceramics, which can be used in the design of materials for electronic applications.

## Figures and Tables

**Table 1:** Lattice parameters and cell volume determined from the Rietveld refinement.

Mole Fraction *x*	Lattice Parameters			V [Å^3^]
	*a*_0_ [Å]	*b*_0_ [Å]	*c*_0_ [Å]	
0.00 [48]	3.9406	3.9406	25.6378	398.1
0.02	3.9303	3.9303	25.6248	395.8
0.04	3.9291	3.9291	25.6127	395.4
0.06	3.9285	3.9285	25.6089	395.2
0.08	3.9259	3.9259	25.5957	394.5
0.10	3.9285	3.9285	25.6059	395.1

**Table 2:** Summary of the average values of the velocities of longitudinal and transverse ultrasonic waves in pure BaBi_2_Nb_2_O_9_ ceramics and those doped with samarium ions.

Mole Fraction *x*	BaBi_2−*x*_Sm*_x_*Nb_2_O_9_	
	*V_L_* [m/s]	*V_T_* [m/s]
0.00 [54]	3647.7	2187.5
0.02	4383.6	2585.2
0.04	4572.4	2569.0
0.06	4527.8	2579.3
0.08	4585.4	2582.6
0.10	4539.2	2569.2

**Table 3:** Results of the mechanical properties of BaBi_2_Nb_2_O_9_ ceramics doped with Sm^3+^ ions.

*x* (Sm)	Poisson’s Ratio, *μ*	Young’s Modulus, *E* [GPa]	Modulus of Stiffness, *G* [GPa]
0.00 [57]	0.219	76.07	31.20
0.02	0.233	119.51	48.45
0.04	0.269	119.06	46.90
0.06	0.229	127.71	51.94
0.08	0.268	123.35	48.65
0.10	0.264	114.23	45.18

**Table 4:** Changes in the maximum electrical permittivity and temperature Tm induced by the modification with samarium ions in the bismuth-oxide layers of BaBi_2_Nb_2_O_9_ ceramics.

*x* (Sm)	*T*_m_ [K] (100 [kHz])	*ε*_max_
0.00 [48]	456.0	406.5
0.02	459.2	525.6
0.04	449.8	437.6
0.06	436.7	424.2
0.08	436.3	451.8
0.10	437.7	508.7

**Table 5:** The diffuseness parameter of the ceramic doped with samarium ions substituted in the bismuth-oxide layers of BaBi_2_Nb_2_O_9_ ceramic.

*x* (Sm)	BaBi_2−*x*_Sm*_x_*Nb_2_O_9_
	*γ*
0.00 [48]	1.45
0.02	178
0.04	177
0.06	188
0.08	189
0.10	190

**Table 6:** Compilation of values for the temperature difference Δ*T*_m_ and the maximum electrical permittivity difference Δε.

*x* (Sm)	BaSm*_x_*Bi_2−*x*_Nb_2_O_9_	
	Δ*T*_m_ [K]	Δ*ε*_max_
0.00 [48]	92.59	68.0
0.02	99.70	50.3
0.04	98.31	47.9
0.06	117.31	66.5
0.08	106.01	61.5
0.10	64.21	92.4

## Data Availability

The original contributions presented in the study are included in the article, further inquiries can be directed to the corresponding author.
